# Why Hoop Tension Matters: A Biomechanical Perspective on Medial Meniscus Posterior Root Tears—A Narrative Review

**DOI:** 10.3390/bioengineering12060638

**Published:** 2025-06-11

**Authors:** Man Soo Kim, Yong In, Hyungtae Kim, Juyoung Jeong, Sueen Sohn

**Affiliations:** 1 Department of Orthopaedic Surgery, Seoul St. Mary’s Hospital, College of Medicine, The Catholic University of Korea, Seoul 06591, Republic of Korea; kms3779@naver.com (M.S.K.); iy1000@catholic.ac.kr (Y.I.); 2 Department of Orthopaedic Surgery, Inje University Sanggye Paik Hospital, College of Medicine, Inje University, Seoul 01757, Republic of Korea; mofi1027@naver.com (H.K.); jeong930118@naver.com (J.J.)

**Keywords:** medial meniscus, posterior root, biomechanics, hoop tension, tensile strength

## Abstract

This narrative review aims to provide an in-depth understanding of the biomechanical consequences of medial meniscus posterior root tears (MMPRTs), with a particular focus on the role of hoop tension in meniscal function. By revisiting fundamental principles such as load transmission, contact mechanics, and structural stabilization, this review elucidates how MMPRTs compromise both the integrity and function of the knee joint. The disruption of hoop tension is analyzed across various tear patterns, and through a synthesis of biomechanical experiments, the superiority and necessity of anatomical structural restoration over conservative management or meniscectomy are emphasized. A comprehensive grasp of these biomechanical foundations offers a critical perspective on the pathomechanics of MMPRTs and serves as a basis for more rational, evidence-based surgical decision-making in clinical practice.

## 1. Introduction

Meniscal injuries are common and clinically significant conditions that can impair knee function and predispose patients to long-term joint degeneration. The incidence of meniscal injury in the United States ranges from 0.6 to 0.7 per 1000 person years [1]. In young and physically active populations, meniscal tears frequently occur as isolated injuries or in conjunction with ligamentous damage, often due to sports-related trauma. Meniscal injuries typically result from torsional trauma to a flexed knee during pivoting sports activities, complex ligamentous injuries such as those involving the anterior cruciate ligament (ACL), or degenerative changes typically associated with age-related joint degeneration. [2,3]. Among various types of meniscal injury, posterior root tears of the medial meniscus have gained increasing attention due to their important biomechanical consequences.

Medial meniscus posterior root tears (MMPRTs) have been reported to account for approximately 10–21% of all meniscal tears. In the past, these lesions were frequently underdiagnosed and undertreated. However, over the past decade, advances in diagnostic imaging and growing clinical awareness have revealed the true burden of MMPRTs—often referred to as a “silent pandemic” [4,5,6]. When left untreated, MMPRTs result in substantial alterations in knee biomechanics, leading to progressive degeneration of both the meniscus and the articular cartilage and, ultimately, contributing to the development of knee osteoarthritis. In one study, MMPRTs were identified in 78.2% of knees that underwent total knee arthroplasty for ostoarthritis [7]. Emerging evidence has shown that MMPRTs may contribute to subchondral insufficiency fractures and early joint degeneration, even in the absence of advanced osteoarthritis [8,9]. Despite their clinical importance, past treatment strategies for MMPRTs often consisted of conservative management, partial meniscectomy, or, in advanced cases, total knee arthroplasty. However, more recently, there has been a growing emphasis on knee-salvageable treatments, and both research and clinical applications of these treatments have expanded considerably [10,11,12].

Mounting evidence suggests that root repair yields superior clinical and radiographic outcomes compared to conservative treatment or partial meniscectomy [13,14]. Nonetheless, controversy remains, and conservative treatment remains a not infrequent choice in clinical practice [15]. This is largely due to the multifactorial nature of treatment decision-making, which must account for patient age, activity level, severity of osteoarthritis, lower limb alignment, and general medical comorbidities [16,17]. Moreover, variability in patient comprehension and adherence to rehabilitation protocols may influence surgical outcomes, often complicating the alignment between patient expectations and actual results. Several additional factors also contribute to the frequent selection of conservative treatment, including its short-term effectiveness, the technical learning curve associated with arthroscopic root repair, the relative familiarity and simplicity of alternative surgical procedures, and the delayed onset of outcomes of biomechanical deterioration seen in some patients [15,17].

Despite these multifactorial reasons that often lead to conservative treatment, a meaningful understanding of meniscal biomechanics is essential for recognizing the necessity of surgical repair and supporting its integration into clinical practice. Previous review articles have primarily emphasized the diagnosis and treatment of MMPRTs, with biomechanical aspects addressed briefly [18,19]. These highlight the need for a more comprehensive understanding of biomechanical principles at this point, not only to underscore the value of structural restoration over conservative management but also to reinforce more informed and evidence-based treatment decisions. Therefore, this review aims to explore the anatomical and functional characteristics of the medial meniscus, the fundamental concept of hoop tension, and current biomechanical perspectives on MMPRTs. To guide this review, we pose the following key questions: (1) How does the restoration of hoop tension serve as the fundamental biomechanical principle underlying the treatment of MMPRTs? (2) What are the biomechanical consequences of an MMPRT?

## 2. Medial Meniscus

### 2.1. Anatomy

The medial meniscus is a semilunar fibrocartilaginous structure interposed between the medial femoral condyle and the medial tibial plateau, serving a crucial role in load transmission, shock absorption, and joint stabilization within the knee joint [20,21]. Anatomically, it is broader posteriorly and narrower anteriorly and less mobile than its lateral counterpart due to its robust capsular and ligamentous attachments [22]. Anatomical characteristics of the medial meniscus are presented in Table 1 [22,23,24]. This morphology allows it to act as a spacer that enhances the congruency between the convex femoral condyle and the relatively flat tibial plateau, thereby increasing the joint contact area and reducing peak contact stress during axial loading [25].

Histologically, the medial meniscus is composed of approximately 70% water and 30% organic matrix, of which collagen accounts for 75% of the dry weight. Type I collagen is predominant throughout the tissue, providing tensile strength, while minor amounts of type II, III, V, and VI collagen exist depending on the region. The superficial layer consists of randomly arranged collagen fibers forming a smooth articulating surface to reduce friction [26,27]. In contrast, the deep layer consists of circumferentially arranged type I collagen fibers, which are critical in converting compressive axial loads into hoop stresses—a core biomechanical function of the meniscus. Additionally, radial tie fibers interconnect these circumferential bundles and function to resist longitudinal splitting [28,29,30]. Proteoglycans, although representing <1% of the ECM, are highly hydrophilic and are essential for fluid retention and compressive resistance. Aggrecan is the dominant proteoglycan, supported by minor molecules like decorin, biglycan, and fibromodulin, which help maintain viscoelasticity [31,32,33]. Cellularity varies by zone: the peripheral vascularized (red-red) zone predominantly contains fibroblast-like cells, while the avascular inner (white-white) zone is occupied by fibrochondrocytes, which are metabolically adapted to hypoxic conditions [26,34,35].

The vascular supply of the medial meniscus is primarily derived from a perimeniscal capillary plexus. This vascularization is limited to the outer 10–30% of the meniscus periphery, with vessels penetrating 2–3 mm radially into the stroma. However, the anterior and posterior roots receive blood supply through endoligamentous capillary loops, providing some nutrition even in deeper portions. The vascular fringe further contributes superficial blood supply to the femoral and tibial surfaces of the menisci, up to 50% of the tibial surface and 100% of the femoral surface in the anterior horn. In the posterior horn, this drops to 25–40% coverage, reflecting the relatively lower healing potential of tears in this region [36,37].

This limited vascularity, particularly in the inner and posterior regions, imposes critical biological constraints on healing. The reduced perfusion not only limits the delivery of essential growth factors and cytokines but also creates a hypoxic microenvironment that impairs cell metabolism and extracellular matrix synthesis. Moreover, the imbalance between pro-inflammatory cytokines and matrix-degrading enzymes further suppresses the regenerative capacity of the tissue, ultimately compromising the biological success of meniscal repair [38].

### 2.2. Functions and Biomechanics of Normal Medial Meniscus

The menisci play an essential role in protecting the tibiofemoral (TF) joint and optimizing joint function. Among their multiple contributions, the most biomechanically significant include load transmission, shock absorption, and joint stabilization. Anatomically, the femoral condyles are convex, while the medial tibial plateau is slightly concave and the lateral tibial plateau is convex. The menisci compensate for this morphological mismatch by filling the void and facilitating congruent articulation. During knee extension, the medial meniscus transmits approximately 40% to 50% of the load, whereas the lateral meniscus transmits 65% to 70% [39,40,41]. In flexion, the contribution of the menisci becomes more prominent, with the lateral meniscus transmitting up to 90% of the load. In the presence of meniscal tears or defects, the TF joint contact area is reduced, and contact pressures are markedly increased. Specifically, horizontal cleavage tears can elevate contact pressures by up to 70%, and radial tears may increase pressures by 70% to 110% [42,43,44]. Complete meniscal root tears result in contact pressure elevations comparable to those observed after total meniscectomy [45].

The meniscus contributes significantly to the shock absorption function of the knee joint, mitigating the effects of both impact and repetitive loads. This function is facilitated by the tissue’s intrinsic viscoelastic properties and interstitial fluid flow, which generate frictional drag forces that dissipate mechanical energy during gait and landing activities [46]. Voloshin and Wosk’s in vivo study using accelerometry demonstrated that knees with either pain or meniscectomy exhibited approximately 20% lower shock attenuation compared to healthy knees, implicating meniscal integrity as crucial for preserving the joint’s natural damping capacity [47]. Complementing this, another in vitro porcine model revealed that both impact forces and repetitive load absorption capability declined significantly following meniscal resection, by up to 68% after total meniscectomy. These findings underscore the meniscus’s central role in maintaining mechanical homeostasis through dynamic shock absorption [46].

The medial meniscus contributes more substantially to joint stabilization than the lateral meniscus, primarily due to differences in mobility and anatomical attachments. While the lateral meniscus is relatively mobile, accommodating the greater anteroposterior translation of the lateral femoral condyle, the medial meniscus is more constrained due to firm meniscocapsular attachments and its tethering to the deep medial collateral ligament. This limited mobility allows it to function as a secondary stabilizer, particularly in the anteroposterior direction. In knees with intact ACL, medial meniscectomy does not significantly alter anterior tibial translation, suggesting that primary ligamentous structures adequately maintain stability [48]. However, in ACL-deficient knees, the stabilizing role of the medial meniscus becomes critical. Allen et al. demonstrated that, under an anterior tibial load, the medial meniscus experiences up to a 197% increase in force at 60° of flexion. Following medial meniscectomy in ACL-deficient knees, anterior tibial translation increases significantly, ranging from 2.2 mm at full extension to 5.8 mm at 60° of flexion, further confirming the medial meniscus as a key secondary stabilizer [49].

## 3. Hoop Tension

**Figure 1:**
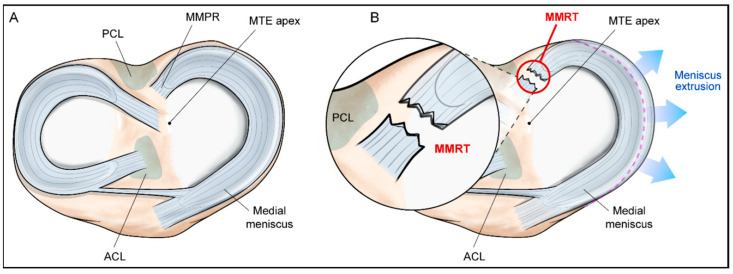
(**A**) Illustration of the intact medial meniscus and adjacent anatomical structures; (**B**) representation of a medial meniscus posterior root tear, demonstrating loss of hoop tension and associated meniscal extrusion. Reproduced from Moon et al., 2023 [19], licensed under CC BY 4.0.

Hoop tension is a critical biomechanical phenomenon by which the meniscus resists radial extrusion and efficiently distributes axial loads across the TF joint. It is generated as compressive forces applied to the meniscus are converted into circumferential tensile stresses, maintaining joint congruity and protecting the articular cartilage from localized overloading (Figure 1). When axial forces, such as those encountered during weight bearing, are applied to the knee joint, the meniscus attempts to disperse the load radially. However, because the meniscal horns are securely anchored to the tibial plateau, the radial load is transformed into a circumferential traction force. Although the circumferential collagen fiber arrangement and tissue hydration maintained by proteoglycans are fundamental, they alone do not fully explain the stability of hoop tension under dynamic loading. Rather, hoop tension is sustained through a complex interplay of structural and biomechanical factors, including collagen crosslinking, peripheral anchoring, viscoelastic properties, and hydraulic support mechanisms.

### 3.1. Structural and Material Contributors

(1) Primary structures: The meniscus exhibits a highly specialized arrangement of collagen fibers, which are organized into three distinct layers. The superficial layer consists of a delicate meshwork of randomly oriented fibrils that cover the femoral and tibial surfaces. Beneath this, the lamellar layer is composed of tightly packed collagen fibril bundles, which are predominantly arranged in a radial direction at the anterior and posterior horns and intersect at various angles in other regions. The central layer forms the main bulk of the meniscus, where collagen fibril bundles are arranged circumferentially. This circular organization of collagen fibers constitutes the structural basis for the generation of hoop tension in the meniscus [50]. Beyond the spatial arrangement of collagen fibrils, the biomechanical strength of the meniscus is also influenced by the degree of intermolecular crosslinking among collagen fibers. Enzymatic crosslinking, primarily mediated by lysyl oxidase activity, enhances the tensile properties of collagen fibrils, contributing to the maintenance of hoop tension under physiological loading conditions. Strong crosslinking increases the tissue’s resistance to fiber sliding and rupture, ensuring the integrity of the circumferential tension system [51].

(2) Macrostructural constraint: The structural anchoring of the meniscus to surrounding tissues is another critical factor in preserving hoop tension. The meniscal horns are firmly attached to the tibial plateau via robust fibrocartilaginous insertions, while the peripheral meniscal margins are connected to the joint capsule through the meniscocapsular attachments. These anchoring structures prevent the radial extrusion of the meniscus under load, thereby sustaining the circumferential hoop stress necessary for effective load distribution [52].

(3) Matrix material property: Proteoglycans within the meniscus play a crucial role in maintaining tissue hydration and enhancing compressive resistance. Their negatively charged glycosaminoglycan (GAG) chains attract and retain water molecules, generating an osmotic swelling pressure that contributes to the mechanical stiffness and durability of the tissue [53]. Upon axial loading, the retained interstitial fluid creates internal hydraulic pressure, which assists in distributing compressive forces throughout the matrix. This fluid-supported load sharing reduces localized stress concentrations on collagen fibrils and supports the maintenance of hoop tension, thereby minimizing the risk of structural damage under repetitive compressive stresses [54].

(4) Viscoelastic behavior: The meniscus exhibits viscoelastic properties, meaning its mechanical response is both time- and rate-dependent. Under sustained loading, the tissue demonstrates creep (gradual deformation) and stress relaxation (decline in internal stress over time), allowing the meniscus to adapt dynamically to varying mechanical environments. This viscoelasticity not only accommodates load fluctuations but also stabilizes the hoop tension by modulating internal stress redistribution during prolonged weight-bearing activities [55] (Table 2).

### 3.2. Alteration of the Hoop Tension by Tear Type

(1) Horizontal tear

A finite-element analysis (FEA) using a rabbit knee joint model revealed that a horizontal tear resulted in a nonhomogeneous stress distribution within the meniscus, a decrease in axial load transmission by the meniscus, and both increased contact pressure and decreased contact area on the articular surfaces. A horizontal tear could lead to the compression of the superior and inferior leaves, resulting in stress concentration toward the outer edge and a subsequent loss of hoop tension within the meniscus. Interestingly, radial displacement decreased following the horizontal tear, contrary to expectations that hoop tension loss would increase extrusion. This finding could be attributed to the reduced load-bearing function of the meniscus after horizontal tear, which led to diminished compressive load transmission and, consequently, less force-induced deformation, resulting in decreased radial displacement [56]. In a cadaveric study, a 1.5 cm horizontal tear in the posterior third of the medial meniscus, designed to simulate a partial-thickness laminar separation, did not significantly alter peak contact pressure or contact area under axial loading at multiple flexion angles, suggesting that small, localized tears may preserve hoop tension and load distribution [57]. In contrast, Beamer et al. reported that a more extensive full-length horizontal cleavage tear led to approximately a 70% increase in peak contact pressure and a significant reduction in contact area across all tested flexion angles. While neither study directly quantified hoop tension or circumferential stress, the discrepancy in biomechanical outcomes implies that tear morphology—specifically the extent and depth—may critically influence hoop tension disruption. These results support the concept that circumferential collagen fiber integrity is fundamental to meniscal function, and that progressive laminar cleavage may ultimately lead to structural destabilization, even when early-stage tears appear biomechanically stable [44]. Therefore, while horizontal tears are often considered less disruptive to hoop tension than radial tears, emerging evidence suggests that this assumption may not always hold true. Clinicians should carefully assess the extent and depth of the tear, as these morphological characteristics critically influence its clinical and biomechanical consequences.

(2) Radial tear

Radial meniscal tears alter both the magnitude and spatial distribution of contact pressure, contributing to articular cartilage injury. A cadaveric knee study systematically created radial tears involving 30%, 60%, and 90% of the meniscal width at the junction between the body and the posterior horn of the medial meniscus and assessed the dynamic contact mechanics of the knee during simulated gait loading. Only the 90% radial tear condition resulted in a 10–15% increase in mean contact pressure, a 78% increase in peak contact pressure, and a 2.5% reduction in total contact area. Notably, there was a distinct posterocentral shift in the location of peak pressure transmission on the tibial plateau. As a result, regions of the tibial cartilage that are not normally adapted to absorb load (the unconditioned zones) are exposed to increased stress [58]. Prior studies suggest that such load shifting may be more detrimental than pressure elevation alone [59]. This redistribution has important clinical implications, as localized overloading in structurally vulnerable areas may accelerate cartilage damage and osteoarthritic progression even in the presence of modest pressure elevation. Consequently, recognizing and preserving the hoop tension in radial tears may be key to protecting joint integrity and maintaining long-term knee function.

A recent finite-element musculoskeletal simulation study evaluated the biomechanical effects of radial meniscal tears based on their location (anterior horn, midbody, posterior horn) and depth (33%, 50%, 83%). A novel aspect of this study was the quantification of shear stress at the tear margin (the region where the fibrous structure remains intact and continuous). Shear stress at the tear margin increased most prominently, approximately three-fold, in the midbody 83% tear, and, to a lesser extent, in the anterior horn tear, suggesting a greater tendency for tear aggravation in these regions. In contrast, the posterior horn 83% tear showed no significant increase in stress at the tear margin. Instead, the tear end (the region directly adjacent to the radial defect) exhibited a highly localized concentration of stress, distinct from the patterns seen in midbody and anterior horn tears, indicating a different but still elevated risk of tear propagation. The study also confirmed previously reported biomechanical changes, including increased peak contact pressure, a posteromedial shift in load transmission, and increased medial meniscus extrusion. This study further revealed that the total tibial cartilage contact force across the stance phase did not increase with larger radial tears. This finding suggests that the loss of hoop tension may be more sensitively reflected in the spatial distribution and focal concentration of contact pressure on the cartilage surface, rather than in the overall magnitude of load transmitted to the cartilage [60]. Since radial tears can lead to a wide range of biomechanical consequences depending not only on their location and depth but also on the pattern of remaining fiber continuity, further clinical studies are warranted. Clinicians should also recognize that cartilage damage may be exacerbated not merely by an increase in contact pressure but by qualitative alterations in load distribution across the joint surface.

(3) Root tear

A root tear disrupts the meniscal insertion at its tibial attachment, effectively severing the continuity of circumferential collagen fibers. This structural failure compromises the ability of the meniscus to convert axial loads into circumferential hoop stress, leading to a near-complete loss of its load-sharing function. Biomechanical studies have demonstrated that a posterior root tear of the medial meniscus results in contact mechanics comparable to those observed after total meniscectomy, with significantly increased peak TF contact pressures and reduced contact area [61,62,63]. These alterations contribute to accelerated cartilage degeneration and joint instability (Table 3).

## 4. Medial Meniscus Posterior Root Tear

### 4.1. Anatomy of Roots of Medial Meniscus

The posterior root of the medial meniscus anchors it to the posterior intercondylar area of the tibia, just anterior to the posterior cruciate ligament (PCL). The root has a mean footprint area of 30.4 ± 2.9 mm^2^ and lies 9.6 mm posterior, 0.7 mm lateral, and 6.0 mm inferior to the medial tibial eminence apex. This insertion site is critical for translating axial loads into circumferential stresses and preventing medial extrusion. The shiny white fibers of the posterior horn further extend the root’s functional area, comprising up to 60.8% of the overall root footprint. These fibers are closely associated with the PCL insertion, making this area vulnerable during procedures involving tibial tunnel drilling [64]. The anterior root inserts into the anterior intercondylar region, approximately 27–30 mm proximal to the tibial tuberosity, and shows significant anatomic variation. It is composed of a central dense fiber bundle, supplemented by thinner peripheral extensions. These insert anteromedially to the ACL tibial insertion, with an average footprint of 56.3 mm^2^ (central bundle only) and 140.7 mm^2^ when including supplemental fibers. Importantly, the anterior intermeniscal ligament, when present, also connects this root to the lateral meniscus, potentially influencing meniscal kinematics [65].

### 4.2. Definition and Classification

**Figure 2:**
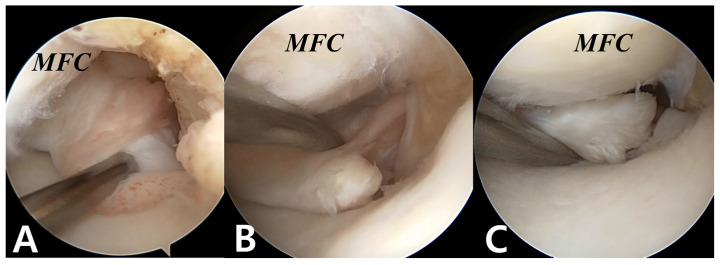
Arthroscopic images of medial meniscus posterior root tears. (**A**) Stable partial tear; (**B**) complete radial tear with advanced scarring at the torn end; (**C**) complete radial tear with a remnant portion of the medial root. MFC: Medial femoral condyle.

An MMPRT is typically defined as a radial tear or avulsion occurring adjacent to the tibial attachment of the posterior horn of the medial meniscus (Figure 2). In their study, LaPrade et al. defined root tears as those located within 9 mm of the tibial attachment of the medial meniscus posterior horn based on biomechanical findings demonstrating that radial tears within this range induce joint contact mechanics similar to those seen in root avulsions [66,67]. Utilizing 71 cases of medial and lateral meniscus root tears, they proposed a morphology-based classification system consisting of five types [66]. Type 1 refers to a partial but stable tear; Type 2 is a complete radial tear within 9 mm of the root attachment; Type 3 describes a bucket-handle tear with concomitant complete detachment of the root; Type 4 is a complex oblique or longitudinal tear extending into the root attachment; Type 5 represents a bony avulsion of the root from the tibial plateau. The most frequent tear morphology was Type 2 (52.1%). Focusing on the posterior medial root, Type 2 tears accounted for 78.4% (29/37), followed by Type 4 (10.8%) and Type 3 (5.4%).

### 4.3. Biomechanical Alteration

(1) Cadaver study

Allaire et al. conducted a foundational study using nine human cadaveric knees and measured TF contact pressures and knee kinematics under axial loading at various flexion angles (0°, 30°, 60°, and 90°) [61]. They found that an MMPRT led to a 25% increase in peak medial contact pressure compared to the intact state, with biomechanical changes resembling those seen after total meniscectomy. The root tear also caused increased external tibial rotation and lateral translation, likely due to compromised structural restraint between the femur and tibia, particularly during flexion. Additionally, meniscal extrusion and enhanced mobility under compressive load allowed lateral displacement of the tibia. These alterations indicate that MMPRTs affect not only contact pressure but also knee joint stability and alignment. A subsequent study using 10 paired fresh–frozen human knees at identical flexion angles corroborated these findings, reporting similarly elevated contact pressures and meniscectomy-like biomechanical patterns in the MMPRT state [63]. Notably, at 60° and 90° of flexion, the total meniscectomy state demonstrated significantly higher peak pressures than the root tear, supporting the notion that a root tear may approximate the biomechanical impact of total meniscectomy, particularly in a flexed position. To further investigate how the location of a radial tear relative to the root influences knee biomechanics, Padalecki et al. conducted tests using six fresh–frozen human knees, in which radial tears were placed 3, 6, and 9 mm medial to the posterior root attachment and evaluated across five flexion angles (0°, 30°, 45°, 60°, and 90°) [67]. All tear conditions led to a significant reduction in contact area and an increase in contact pressure at flexed positions, with the magnitude of these changes increasing as the tear extended further from the root. Interestingly, at 0° of flexion, contact pressures remained similar to the intact state, which is consistent with prior studies. These findings highlight that not only the presence but also the location of a radial tear relative to the root attachment critically determines the degree of meniscal dysfunction, particularly under load-bearing conditions involving flexion. While prior quasi-static studies assessed contact mechanics at discrete flexion angles, a more recent investigation applied simulated gait loading using a robotic system in six cadaveric knees. Under these dynamic loading conditions, MMPRTs significantly increased peak contact stress and decreased contact area during 20–37% and 16–60% of the gait cycle, respectively (*p* < 0.05). These alterations were most pronounced during the early stance phase, when the knee was flexed less than 15°, suggesting that MMPRTs may impair joint loading even at low flexion angles. The position of the contact center remained unchanged in both anterior–posterior and medial–lateral directions [68]. Collectively, these findings reinforce the biomechanical rationale for early surgical repair and possibly suggest a need to limit early postoperative flexion to protect the healing root.

(2) FEA study

Finite-element analysis (FEA) has emerged as a powerful computational tool in orthopaedic biomechanics, offering the ability to simulate complex joint loading scenarios, quantify internal stress distributions, and evaluate surgical techniques under controlled, repeatable conditions. FEA enables a detailed assessment of localized stresses within cartilage and menisci and allows for systematic manipulation of anatomical parameters, tissue properties, and alignment. In the context of MMPRTs, FEA has gained increasing attention as it facilitates in-depth investigation of repair strategies, tear patterns, and malalignment across dynamic gait cycles—conditions that are difficult to replicate experimentally.

Xu et al. [69] evaluated the biomechanical consequences of MMPRTs under gait loading conditions. Finite-element models were constructed using CT and MRI data from a healthy adult knee. The study found that complete radial and oblique tears led to significant increases in medial contact stress (up to 25.3%) and medial meniscus displacement (over 220%), along with a marked reduction in cartilage contact area (over 30%) compared to the intact knee. In another study simulating a static loading condition, peak stress within the medial meniscus increased by 23.6%, indicating impaired axial load redistribution consistent with a loss of hoop tension [70]. Furthermore, MMPRT increased medial compartment loading—defined as the axial force transmitted from the medial femoral cartilage (MFC) to the medial tibial cartilage (MTC)—approximately three-fold compared to the intact state, with maximum von Mises stress increasing more substantially in the MTC (136%) than in the MFC (24.3%). Under dynamic loading conditions, medial compartment forces exceeded a 3:1 ratio relative to the lateral side during peak gait phases, which is substantially higher than the typical 2:1 load distribution observed in normal gait under physiological conditions [71]. Stress on the medial meniscus was significantly elevated throughout 10–50% of the gait cycle, and peak contact pressure on the MTC was increased relative to the intact model. Notably, under equivalent axial loads, dynamic conditions resulted in more severe stress concentrations than static conditions [70].

As expected, the biomechanical impact of MMPRT became more pronounced with increasing varus alignment. Under static loading conditions, analysis across varying lower limb alignments revealed that the MMPRT state resulted in a marked increase in medial compartment peak contact pressure compared to the intact knee. Peak contact pressure progressively increased as varus malalignment worsened. Concurrently, total contact area decreased by up to 46% in the MMPRT condition, with greater reductions observed as varus alignment increased. These changes were accompanied by substantial elevations in focal contact pressure, reflecting a highly uneven and concentrated stress distribution within the medial compartment [72].

The aforementioned FEA studies enable detailed analysis of load distribution and joint mechanics under various conditions. However, current models are typically based on single-subject representations rather than cohorts of patients with osteoarthritis or diverse demographic characteristics. Additionally, they do not account for biological processes such as tissue healing after injury or repair. Future studies should aim to advance finite-element modeling through the development of personalized, patient-specific simulations that account for individual anatomical and alignment variations. Statistical shape modeling (SSM) and related techniques have shown promise in constructing population-based anatomical models, thereby facilitating individualized biomechanical analysis and enhancing clinical applicability. In addition, incorporating time-dependent changes in tissue properties, such as those associated with healing or degeneration, may allow for more clinically relevant longitudinal analyses. While traditional FEA is inherently limited to mechanical evaluation at discrete time points, integration with biological modeling frameworks could further enhance its utility in predicting long-term outcomes following meniscal injury and repair.

(3) Biomechanical restoration following root repair

Cadaveric studies have consistently demonstrated that transtibial pull-out suture repair of MMPRTs improves biomechanical properties, in some cases restoring them to levels that are not significantly different from the intact meniscus [61]. In one study, repair using the transtibial pull-out technique resulted in normalization of contact pressure and area across tested flexion angles. In contrast, another study using the same repair method reported significant reductions in peak contact pressure and increases in contact area at 30° and 60° of knee flexion when compared to the root tear state. However, full restoration to normal levels was not achieved. In particular, at 60° and 90° of flexion, the contact area remained significantly reduced relative to the intact condition, suggesting that root repair only partially restores meniscal function, and that its efficacy may be limited in deeper flexion [63]. In a separate study evaluating radial tears located at varying distances from the root attachment, in situ suture repair effectively normalized contact mechanics in 3 mm and 6 mm tear conditions. However, for tears located 9 mm from the root base, while contact pressure was restored to normal, the contact area remained significantly lower than that of intact knees [67]. Most recently, a dynamic cadaveric gait simulation study demonstrated that transtibial root repair during the gait cycle achieved near-complete restoration of both contact pressure and area, indicating the potential of anatomic repair to restore physiological loading conditions under functional gait [68].

One finite-element analysis study reported that while peak contact pressure remained elevated compared to the intact state following meniscal root repair, meniscal displacement and TF contact area were largely restored to near-normal levels. Although these findings appear favorable, the same study noted a marked stress concentration at the root fixation site, which may represent a potential failure risk or predispose the repair site to accelerated degeneration, particularly under repetitive loading conditions [69]. Similarly, another investigation found improvements in stress distribution within the medial meniscus and medial tibial cartilage (MTC), as well as partial restoration of contact area following repair. However, full biomechanical recovery was not achieved, and stress concentration persisted at the site of repair [70]. Furthermore, when considering the influence of lower limb alignment, biomechanical restoration after root repair was found to be increasingly limited in knees with greater varus alignment, suggesting that complete normalization of joint mechanics may not be attainable in such cases and highlighting the potential need for concurrent correction of varus malalignment at the time of root repair [72].

On the other hand, precise restoration of the meniscal root to its native insertion site is critical during repair, as nonanatomic placement results in inferior biomechanical outcomes, often resembling those of an unrepaired root tear. In a cadaveric biomechanical study, the authors defined a nonanatomic repair as fixation of the meniscal root 5 mm posteromedial to its native anatomic insertion site. This misplacement may often occur in clinical settings where the torn root becomes scarred and adherent to the posteromedial capsule, leading to fixation in the extruded position without sufficient mobilization. While anatomic repair restored approximately 83% of the contact area across 0°, 30°, 60°, and 90° of knee flexion, nonanatomic repair demonstrated only about 56%, a value comparable to the root-deficient state. Similarly, mean contact pressure increased by 12–15% following anatomic repair compared to the intact state, whereas nonanatomic repair produced a 67% increase. Peak contact pressure increased by approximately 25% and 58%, respectively. Notably, at 90° of flexion, significant differences in both mean and peak contact pressures were observed between the two repair states, suggesting that nonanatomic repair may be particularly detrimental under deep flexion loading conditions [62]. These findings were further supported by finite-element analysis. A 5 mm posteriorly malpositioned repair resulted in reduced hoop stress, increased meniscal extrusion, decreased contact area, and altered contact pressures, collectively replicating the biomechanical environment of a root-deficient state. In contrast, a 5 mm anterior repair offered improvements in hoop stress restoration, extrusion control, and contact area recovery. However, meniscal contact occurred in nonphysiological regions and was accompanied by increased suture tension, which may predispose to early failure [73].

## 5. Conclusions

The medial meniscus plays a pivotal role in load transmission, shock absorption, and joint stabilization within the knee joint. Its internal network of circumferential collagen fibers is fundamental to generating and maintaining hoop tension, which not only resists axial loads and prevents radial extrusion but also ensures even distribution of joint forces and protection of the articular cartilage. Beyond fiber orientation, the preservation of hoop tension also relies on factors such as collagen crosslinking, peripheral anchorage, tissue hydration, and viscoelastic behavior. Across various types of meniscal tears, disruption of hoop tension is a common biomechanical consequence, though the extent of functional impairment depends on the size and depth of the lesion. Among these, posterior root tears exert the most profound biomechanical impact, not only increasing tibiofemoral contact pressures but also compromising joint stability. Experimental biomechanical studies have demonstrated that MMPRTs result in elevated and uneven stress distributions and altered load trajectories toward unconditioned cartilage zones. In addition, MMPRTs result in biomechanical disruptions that persist throughout the gait cycle under dynamic loading conditions and also alter joint kinematics. These alterations are biomechanically comparable to those seen after total meniscectomy. Although surgical repair techniques have been shown to partially or even near-completely restore joint contact mechanics, residual abnormalities persist, especially under conditions of deep flexion, maintaining abnormal load patterns that may accelerate osteoarthritis progression.

While meniscal repair has demonstrated superior outcomes over conservative treatment or meniscectomy, these results should not be overly attributed to hoop tension restoration alone. Other contributing factors, such as patient selection, surgical technique, biological healing potential, residual meniscal extrusion, and rehabilitation protocols, all interplay in determining the ultimate success of treatment [74,75,76]. Furthermore, recent advances in regenerative medicine, including tissue engineering, cell-based therapies, and gene therapy, may open new avenues for the treatment of MMPRTs [77,78,79]. Nevertheless, a meaningful understanding of the underlying biomechanical principles provides a critical foundation for establishing and applying evidence-based treatment strategies, particularly in guiding surgical root repair.

## Figures and Tables

**Table 1:** Key anatomical dimensions and structural features of the medial meniscus.

Category	Details
Length	Approx. 45.7 mm
Width (Posterior horn)	12.6–17.4 mm
Width (Midbody)	9.3–12.2 mm
Width (Anterior horn)	7.6–9.0 mm
Thickness	5.2–6.9 mm (wedge-shaped)
Surface Area Coverage	51–74% of medial tibial plateau

**Table 2:** Structural and material contributors to hoop tension.

Subcategory	Key Component	Role
Primary structures	Collagen fiber layers (superficial/lamellar/central or circumferential) Collagen crosslinking	Provides a circumferential tensile framework and resists fiber separation under load
Macrostructural constraint	Meniscal root attachments Meniscocapsular connections	Prevents radial extrusion Preserves the circumferential stress loop
Material composition	Proteoglycans (with GAG [glycosaminoglycan] chains) Interstitial fluid	Maintains hydration and internal pressure to support compressive load sharing
Viscoelastic response	Time- and rate-dependent tissue properties	Modulates internal stress and maintains tension stability during prolonged loading

**Table 3:** Biomechanical consequences by type of meniscal tear.

Tear Type	Research Method	Tear Site	Hoop Tension Disruption	Peak Contact Pressure	Contact Area	Load Shift
Horizontal, localized [57]	cadaver	Posterior 1/3	Minimal or preserved	insignificant	insignificant	n.s.
Horizontal, extensive [44]	cadaver	AH to PH	Severe	↑	↓	suggested
Radial, 30–60% [58]	cadaver	Between body and PH	Mild	insignificant	insignificant	None or mild
Radial, 90% [58]	cadaver	Between body and PH	Severe	↑↑	↓	Posterocentral
Root [62]	cadaver	Posterior root attachment	Near Complete	↑↑	↓↓	n.s.

n.s: not specified; AH: anterior horn; PH: posterior horn.

## Data Availability

The data presented in this study are available in the main article.
